# Fruit Quality Characterization and Comprehensive Evaluation of 30 *Chionanthus retusus* Accessions

**DOI:** 10.3390/metabo15090588

**Published:** 2025-09-03

**Authors:** Muge Niu, Jinnan Wang, Baoqiang Huang, Hui Tian, Maotong Sun, Jihong Li, Jing Ren, Cuishuang Liu

**Affiliations:** 1 College of Forestry, Shandong Taishan Forest Ecosystem State Positioning Observation and Research Station, Key Laboratory of State Forestry and Grassland Administration for Forest Cultivation in the Lower Yellow River, Shandong Agricultural University, Tai’an 271018, China; 2240100021@njfu.edu.cn (M.N.); wjn@sdau.edu.cn (J.W.); sunmt0310@bjfu.edu.cn (M.S.); liujianrui@inspur.com (J.R.); liucuishuang@bjfu.edu.cn (C.L.); 2 College of Forestry and Grassland, Nanjing Forestry University, Nanjing 210037, China; 3 Yishui State-Owned Yishan Forestry Farm, Linyi 276400, China; 13573900627@163.com (B.H.); yslcfh@163.com (H.T.)

**Keywords:** *Chionanthus retusus*, fruit morphology, oil, fatty acid composition, phytosterol, tocopherol, comprehensive evaluation

## Abstract

**Objectives:** Research on kernel oil content and secondary metabolites in *Chionanthus retusus* was conducted to evaluate its potential as an oil crop. **Methods:** Fruits from 30 individual trees were collected to analyze morphological traits, oil content, and the composition of fatty acids, phytosterols, and tocopherols. Correlation, cluster, and principal component analyses were performed on the resulting data. **Results:** The mean fresh fruit weight, dry fruit weight, dry kernel weight, and kernel percentage were 77.02 g, 24.33 g, 12.22 g, and 51.14%, respectively. Kernel oil content averaged 35.83%, comprising seven fatty acids with oleic acid as the predominant component. Total phytosterol content reached 279.58 mg/100 g oil, with β-sitosterol being the major constituent among seven detected sterols. Total tocopherols were 571.13 μg/g oil, dominated by γ-tocopherol, indicating a potential antioxidant capacity. These components may reduce the demand for synthetic antioxidant food additives. A significant positive correlation was observed between kernel dry weight and oil content (r = 0.760, *p* < 0.01), supporting kernel dry weight as a key phenotypic indicator for high-oil breeding. Fruit quality traits did not cluster by geographic origin, whereas secondary metabolite profiles showed origin-based clustering. For breeding oil-producing *C. retusus*, select seeds with superior provenances based on secondary metabolites and cultivate them under optimal conditions to develop varieties with plump fruit, thereby boosting yield. Accessions WS-4 and WS-3 were identified as promising germplasm resources for oil production. **Conclusions:** The abundant oleic acid, β-sitosterol, and γ-tocopherol in *C. retusus* kernels highlight its potential as a woody oilseed crop.

## 1. Introduction

In recent years, the demand for vegetable oils has surged dramatically alongside the advancement of modern industrialization [1,2]. China’s reliance on imported edible oils has reached 70%, significantly exceeding the security threshold. Woody oil crops present distinctive advantages, characterized by “non-competition with grain crops for arable land” and “long-term yields from a single planting.” The “whole-resource utilization” model further enhances their resource efficiency. Consequently, vigorously developing perennial woody vegetable oils represents an effective strategy to alleviate the vegetable oil supply gap [3]. However, most woody oil crops require demanding environmental conditions regarding water and nutrients during cultivation and management. Furthermore, the inherent oxidative stability of different plant oils varies considerably, leading to gradual quality degradation during long-distance transport. Moreover, oil quality is typically associated with storage time, temperature, and conditions. During storage, intrinsic factors (such as fatty acid composition, plant secondary metabolites, moisture content, and processing quality) also significantly affect the oxidation process [4]. To extend oil shelf life, antioxidant additives such as butylated hydroxyanisole (BHA) and butylated hydroxytoluene (BHT) are commonly employed [5]. Long-term use or consumption of these additives may pose potential adverse effects on human health. Therefore, identifying and screening novel woody oil plant species with strong ecological adaptability and high intrinsic antioxidant capacity is crucial to address the current demands of the vegetable oil sector.

Currently, the application value of vegetable oils is typically assessed by measuring indicators such as kernel morphological traits, fatty acid composition, phytosterols, and tocopherols [6,7,8]. Fatty acid composition serves as the primary indicator for evaluating oil quality. Vegetable oils are rich in diverse fatty acids, predominantly unsaturated fatty acids (UFAs), including oleic acid, linoleic acid, and linolenic acid, among others. Among these, linoleic acid and linolenic acid are essential fatty acids (EFAs) that must be obtained through dietary intake [9,10], playing crucial roles in physiological functions and growth and development [11]. Studies demonstrate that polyunsaturated fatty acids (PUFAs) can reduce cholesterol levels, regulate blood lipids, decrease blood viscosity, and enhance immunity [12]. Specifically, oleic acid lowers total blood cholesterol and harmful low-density lipoprotein (LDL) cholesterol while maintaining beneficial high-density lipoprotein (HDL) cholesterol levels, thereby preventing or ameliorating cardiovascular and cerebrovascular diseases [13,14]. Linolenic acid, eicosapentaenoic acid (EPA), and docosahexaenoic acid (DHA) play key roles in enhancing brain cell activity and the development of the retina and neural tissues [15,16]. Furthermore, UFAs such as linoleic acid and linolenic acid also enhance immune function, reduce pro-inflammatory factor production, prevent infarction, and inhibit allergic responses [17,18].

Phytosterols and tocopherols are important secondary metabolites and nutritional quality indicators, significantly contributing to an oil’s antioxidant capacity [8]. Phytosterols, structurally analogous to cholesterol, are essential biomolecules for human health that must be acquired from food [19,20,21]. Vegetable oils and cereals represent their optimal natural dietary sources [22]. Research indicates that phytosterols can reduce cardiovascular disease risk by inhibiting cholesterol absorption [23,24] and possess both anti-inflammatory and anti-cancer properties [25,26]. Tocopherols are essential fat-soluble compounds that require dietary intake [27]. Among natural tocopherols, the α-form exhibits the highest absorption and metabolic efficiency in humans, while the γ-form demonstrates the strongest antioxidant capacity [28]. Tocopherols can reduce the risk of cancer, cardiovascular diseases, coronary heart disease, neurological disorders, and pulmonary diseases [29,30,31] and also function in delaying aging and protecting against ultraviolet (UV) damage [32,33,34].

Currently, an increasing number of novel woody oil plants are gaining attention, such as yellowhorn (*Xanthoceras sorbifolia*) [35], sea buckthorn (*Hippophae rhamnoides*) [36], and Shantung maple (*Acer truncatum Bunge*) [5]. Chinese fringetree (*Chionanthus retusus* Lindl. & Paxton), belonging to the genus *Chionanthus* within the Oleaceae family, is typically a deciduous shrub or small tree. It is widely distributed in subtropical and temperate regions of China, with some populations also found on the Korean Peninsula and in Japan [37]. *C. retusus* exhibits strong tolerance to drought, salinity, and waterlogging, making it an excellent native species for greening barren mountains and urban streets [38]. Studies indicate its kernels possess an oil content reaching 36.5% [39], classifying it as a high-oil woody plant. However, research on its kernel oil yield, fatty acid profile, and secondary metabolite composition remains limited. Investigating the oil content and secondary metabolites in *C. retusus* kernels is essential to evaluate its potential as a novel woody oil crop. This study presents the first systematic integrated analysis of variations in key morphological characteristics, oil content, and differences in the content and composition of core nutritional components (fatty acid composition, phytosterols, and tocopherols) in the fruits of diverse *C. retusus* germplasms. Through comprehensive analysis of multidimensional indicators (morphology, content, quality components), including correlation analysis, cluster analysis, and comprehensive evaluation, this research provides an in-depth characterization of the oil properties and nutritional value inherent in *C. retusus* germplasm resources. The findings aim to provide crucial scientific evidence for the precise selection of high-yielding, superior-quality, and nutrient-rich *C. retusus* varieties specifically cultivated for edible oil production. This research will significantly propel the scientific and sustainable development of China’s oil-oriented *C. retusus* industry.

## 2. Materials and Methods

### 2.1. Plant Materials

Shandong Province is the main distribution area of ancient *C. retusus* trees in China. Therefore 30 superior and ancient trees (Wild Resources) of *C. retusus* with high yield and vigorous growth were selected for these analyses. The trees were growing in the cities of Anqiu, Yinyuan, Qingzhou, Tai’an, and Zibo in Shandong Province and in the city of Chengde in Hebei Province (Table 1). In late August 2020, mature fruits (approximately 2 kg per grafted germplasm) were collected following the transition of peel color from green to purple-black. The fruits were picked randomly from different infructescences in the trees’ crowns to ensure that the samples were representative. First, the fruit morphology parameters were measured (fresh weight, fruit width, and fruit length), and then the pulp was removed. Seeds of each accession were oven-dried (BOXUN, Shanghai, China GZX-9070MBE) to constant weight at 65 °C. The kernels, dried in an oven at 65 °C, were considered to have reached constant weight when two consecutive mass measurements, taken at 1 h intervals, showed a change of less than 0.1%. At this point, the average moisture content was 22.72%.

### 2.2. Fruit Morphology

For measurements of morphological parameters, 300 undamaged fruits were randomly selected from each *C. retusus* accession. The fresh weight of fruit was determined as the weight of 100 seeds, accurate to 0.001 g. This measurement was repeated three times. After removing the pulp from the fruit, the grains were dried at 65 °C and the weight of 100 grains was determined, accurate to 0.001 g. This measurement was repeated three times. The kernels were then removed from the seeds, and the weight of the kernels and seed shells were separately determined for 100 grains, accurate to 0.001 g. This measurement was repeated three times. Fruit characteristics (fruit length, FL; fruit width, FW; fruit shape index, FSI) and kernel characteristics (grain length, GL; grain width, GW; shell thickness, ST) were measured to the nearest 0.001 mm. Fruit shape index (FSI) is the ratio of fruit length (FL) to fruit width (FW). Kernel percentage (Kp) was calculated as the ratio of kernel dry biomass to grain dry biomass.

### 2.3. Oil Extraction

Oil was extracted using the Soxhlet method according to the Chinese National Standard [40]. For each *C. retusus* accession, approximately 5 g kernels were crushed into a powder and then subjected to Soxhlet extraction using petroleum ether (boiling point 30–60 °C) as the solvent at 60 °C for 8–10 h. After solvent evaporation, the flask containing oil was dried at 105 °C, cooled in a desiccator, and reweighed.

### 2.4. Fatty Acid Determination

Fatty acid composition was determined by gas chromatography (GC) according to the Chinese National Standard [41]. Saponification of fat and methyl esterification of fatty acids were conducted by adding 8 mL 2% sodium hydroxide methanol solution to the fat extract. The mixture was refluxed in a water bath at 80 °C until the oil droplets disappeared; then, 7 mL 15% boron trifluoride methanol solution was added and the mixture was refluxed for 2 min. It was then rapidly cooled to room temperature; 10–30 mL n-heptane was added, and the mixture was shaken for 2 min. Saturated sodium chloride solution was added, followed by static layering. Then, 3–5 g of anhydrous sodium sulfate was added to approximately 5 mL of the n-heptane extraction supernatant solution. The mixture was shaken for 1 min, followed by static layering for 5 min. Finally, the upper layer of the solution was transferred into an injection vial for determination. The fatty acid methyl esters obtained from each *C. retusus* oil sample were analyzed using an Agilent 7890B gas chromatograph (GC) (Agilent Technologies, 7890A-5975CMS, Santa Clara, CA, USA) fitted with a flame ionization detector and equipped with a DM-2560 capillary column (100 m × 0.25 mm i.d., 0.2 μm film thickness). The injector and detector were programmed at 100 °C, increasing at 10 °C min^−1^ to 180 °C, then at 1 °C min^−1^ to 200 °C, and then at 4 °C min^−1^ to 230 °C for 10.5 min. The flow rate of the carrier gas (nitrogen) was 1.0 mL min^−1^ and the split ratio was 1:100. Fatty acids were identified by comparing retention times with those of standard samples (Supelco 37 FAME mix, Supelco, Bellefonte, PA, USA) and their percentage was calculated according to the area of each peak.

### 2.5. Phytosterols Determination

Phytosterols were determined using a GC according to the Chinese National Standard [42]. The oil sample was saponified with ethanolic potassium hydroxide solution. The unsaponifiable sterol fraction was separated by thin-layer chromatography on a sheet of aluminum foil coated with a thin layer of alumina. Separation and quantification of the silanized sterol fraction were carried out by capillary GC on an Agilent 7890B (Agilent Technologies, 7890A-5975CMS, USA) instrument equipped with an SE-45 capillary column (length, 50 m; i.d.,0.25 mm; film thickness, 0.1 μm). The working conditions were as follows: injector at 320 °C; initial column temperature at 240 °C, increasing at 4 °C min^−1^ to 255 °C; sample injection volume, 1 μL; flow rate, 36 cm/s; split ratio 1:20; and carrier gas, hydrogen. Phytosterols were identified by comparing retention times with those of standard samples (Betulin, Supelco).

### 2.6. Tocopherol Determination

The content and composition of tocopherols were determined using liquid chromatography according to the Chinese National Standard [43]. Each *C. retusus* kernel sample was ground into a powder, then 0.2 g was placed in a test tube before adding 0.05 g vitamin C and 4 mL 80% ethanol solution. The mixture was shaken and mixed thoroughly, then subjected to ultrasonic treatment (SHENHUATAI, PS40A) in a low-temperature water bath for 30 min before adding 8 mL n-hexane solution. The mixture was then centrifuged and the supernatant was passed through a 0.22 μM organic phase filter membrane. Subsequently, analysis was performed using high-performance liquid chromatography (HPLC) (Agilent Technologies, Agilent 1260). Tocopherols were identified by comparing retention times with those of standard samples (Betulin, Supelco).

### 2.7. Statistical Analyses

Data on fruit characteristics, kernel characteristics, yield, kernel percentage (Kp), oil content, sterols, and tocopherol, as well as fatty acid and sterol composition, were analyzed using IBM SPSS 19.0 software (IBM Corp., Armonk, NY, USA). Statistical analyses included analysis of variance (ANOVA) followed by Tukey’s honestly significant difference (HSD) test for multiple comparisons, correlation analysis, and cluster analysis. Principal component analysis (PCA) was performed using SAS 9.2 software (SAS Institute, Cary, NC, USA). Cluster heatmaps were constructed using TBtools-II V2.225 software. The data presented in the above experimental analysis were obtained from three independent replicates and are reported as the mean ± standard deviation.

## 3. Results

### 3.1. Fruit Morphological Diversity in Chionanthus retusus

Morphological characteristics serve as important phenotypic indicators for evaluating phenotypic diversity in germplasm and screening superior clones for oil production [44,45]. The fruit morphological parameters (fresh weight, FL, FW, FSI), kernel traits (dry weight, GL, GW, ST, GL/ST), and yield indices (kernel dry weight, shell dry weight) of 30 *C. retusus* germplasms are summarized in Table 2. Significant differences (*p* < 0.05) were observed among the germplasms for all morphological parameters. The average fruit fresh weight was 77.02 g (ranging from 47.42 g to 121.30 g, CV = 27.15%). The mean fruit dry weight was 24.33 g (14.33 g to 36.07 g, CV = 26.75%), while the mean kernel dry weight was 12.22 g (7.751 g to 16.67 g, CV = 26.52%). The average fruit length (FL), fruit width (FW), grain length (GL), and grain width (GW) were 12.46 mm, 9.98 mm, 10.85 mm, and 6.71 mm, respectively. The coefficient of variation (CV) for fruit morphological traits across different germplasms ranged from 9.53% to 32.42%. This substantial variation offers great potential for the efficient selection of superior germplasm.

### 3.2. Oil Content and Fatty Acid Composition of C. retusus Kernels

Oil content is a key indicator for evaluating the potential of kernels as an oilseed crop, with higher content reducing extraction costs. Analysis of oil content in kernels from 30 *C. retusus* germplasms (Table 3) revealed a mean of 35.83%, ranging from 28.30% (S-3) to 47.50% (WS-5), with a coefficient of variation (CV) of 15.81%. The lowest value (S-3) showed no significant difference from B-1 (28.90%), WA-1 (29.00%), and S-2 (29.20%). This variation may be attributed to the genetic diversity among germplasms [45].

Fatty acid composition is a critical parameter for assessing oil quality. Seven major fatty acids were detected in the kernel oil of the 30 germplasms (Table 2). Oleic acid (C18:1) was predominant (49.53–59.67%, mean 54.18%), followed by linoleic acid (C18:2) (15.33–30.35%, mean 23.57%), linolenic acid (C18:3) (5.00–13.06%, mean 9.57%), palmitic acid (C16:0) (0.41–2.09%, mean 1.29%), stearic acid (C18:0) (0.45–1.88%, mean 1.12%), gondoic acid (C20:1) (0.25–0.40%, mean 0.33%), and arachidic acid (C20:0) (0.12–0.21%, mean 0.14%). Notably, arachidic acid (C20:0) was only detected in germplasms from the WA, WS, and S groups.

Unsaturated fatty acids (UFAs), known for their health benefits, are key quality markers for vegetable oils. Based on double-bond count, fatty acids were categorized as saturated (SFA), monounsaturated (MUFA), or polyunsaturated (PUFA). The mean SFA content was 2.41% (range: 1.00–3.64%, CV = 12.02%), MUFA was 54.51% (49.87–60.00%, CV = 6.17%), and PUFA was 33.23% (25.01–42.74%, CV = 10.22%) (Table 3). UFA predominated in *C. retusus* kernel oil, accounting for 79.55–95.84% of total fatty acids, highlighting its potential as a high-quality oil crop. Germplasms WS-2, WS-3, and WS-5 showed notably high oleic acid content, making them valuable resources for developing high-oleic cultivars. The coefficients of variation (CV) among the indices ranged from 5.84% to 22.22%, revealing significant variation in kernel fatty acid content across different germplasms.

### 3.3. Phytosterol and Tocopherol Content and Composition in C. retusus Kernel Oil

To characterize secondary metabolites in *C. retusus* kernels, we investigated the content and composition of phytosterols and tocopherols (Table 4 and Table 5). Results revealed significant differences (*p* < 0.05) in total phytosterol content among the 30 germplasms (Table 4), ranging from 225.71 mg/100 g (S-5) to 360.59 mg/100 g (WA-2), with a mean of 279.58 mg/100 g (CV = 15.68%). Phytosterols are structurally diverse, with β-sitosterol, campesterol, and stigmasterol being the most prevalent in plants [21,23]. Capillary gas chromatography identified seven phytosterols in the oil. β-Sitosterol predominated (mean 188.16 mg/100 g; range 154.78 mg/100 g (WS-10)–240.67 mg/100 g (WA-2)), followed by β-sitostanol (mean 36.88 mg/100 g; range 31.66 mg/100 g (B-2)–49.65 mg/100 g (T-8)). Other components included campestanol (12.09–42.90 mg/100 g), campesteranol (10.07–19.30 mg/100 g), campesterol (10.99–22.95 mg/100 g), and Δ5-avenasterol (2.26–9.87 mg/100 g). The overall coefficient of variation (CV) for phytosterol content ranged from 12.54% to 27.22%. Substantial differences in kernel phytosterol levels were revealed among different germplasms.

Total tocopherol content also showed significant inter-germplasm variation (*p* < 0.05) (Table 5). The mean total tocopherol content was 571.13 µg/g (range: 480.94 µg/g (Z-1)–654.22 µg/g (T-10); CV = 10.14%). HPLC analysis detected four tocopherol isomers: γ-tocopherol was dominant (520.97 µg/g), followed by α-tocopherol (33.76 µg/g), with δ-tocopherol and β-tocopherol present at 9.16 µg/g and 7.24 µg/g, respectively. The coefficient of variation (CV) for total tocopherols ranged from 9.47% to 31.77%. Significant differences in total tocopherol content were observed among different germplasm kernels.

### 3.4. Correlation Analysis of Fruit Morphological Traits, Fatty Acid Composition, and Oil, Phytosterol, and Tocopherol Content in C. retusus

**Figure 1:**
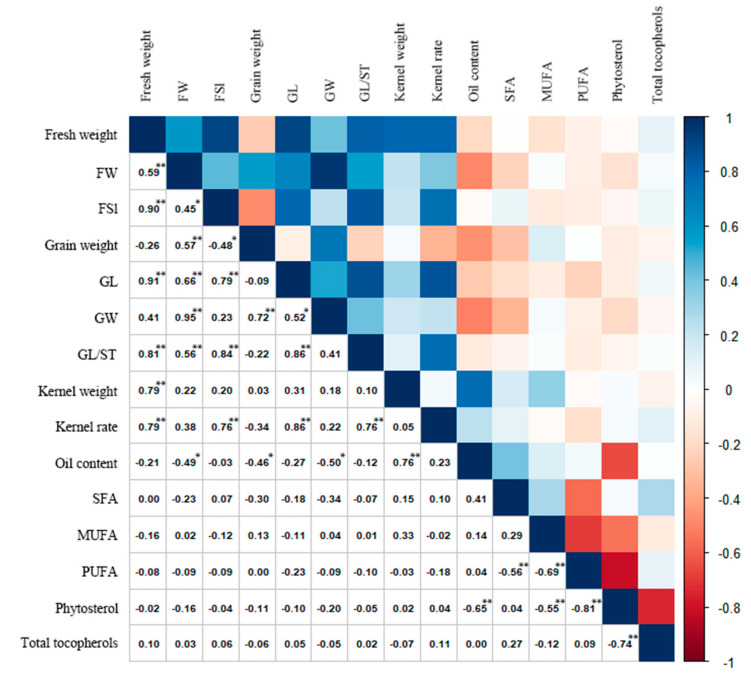
Spearman’s correlation coefficients calculated from pair-wise comparisons between fruit morphology, oil content, phytosterol content, tocopherol content, and saturated, monounsaturated, and polyunsaturated fatty acid content in *C. retusus* accessions. FW, fruit width; FSI, fruit shape index; GL, grain length; GW, grain width; GL/ST, grain length/shell thickness; SFA, saturated fatty acid; MUFA, monounsaturated fatty acid; PUFA, polyunsaturated fatty acid. * *p* < 0.05; ** *p* < 0.01.

Subsequent analysis was performed on 15 selected key indicators. Spearman correlation coefficients of the fruit morphological parameters, oil content, SFA, MUFA, PUFA, and total phytosterol content across 30 *C. retusus* germplasm accessions are presented in Figure 1. Kp showed highly significant positive correlations with fruit fresh weight (r = 0.790, *p* < 0.01), fruit shape index (FSI; r = 0.758, *p* < 0.01), grain length (GL; r = 0.860, *p* < 0.01), and GL/shell thickness (GL/ST; r = 0.760, *p* < 0.01). Kernel dry weight was significantly positively correlated with fruit fresh weight (r = 0.790, *p* < 0.01) and oil content (r = 0.760, *p* < 0.01). Fruit fresh weight exhibited highly significant positive correlations with fruit width (FW; r = 0.589, *p* < 0.01), FSI (r = 0.902, *p* < 0.01), GL (r = 0.907, *p* < 0.01), GL/ST (r = 0.810, *p* < 0.01), kernel dry weight (r = 0.794, *p* < 0.01), and Kp (r = 0.790, *p* < 0.01). These results indicate that fruit fresh weight serves as a key phenotypic indicator for selecting germplasms with high kernel dry weight and Kp in *C. retusus* breeding programs, while kernel dry weight is a critical indicator for screening high-oil-content germplasms.

Significant associations existed among oil, sterol, and tocopherol content. Total oil content correlated negatively with total phytosterol content (r = −0.650, *p* < 0.01) but positively with kernel dry weight (r = 0.760, *p* < 0.01). Highly significant negative correlations (*p* < 0.01) were observed between SFA and PUFA (r = −0.560), MUFA and PUFA (r = −0.690), MUFA and total phytosterols (r = −0.550), and PUFA and total phytosterols (r = −0.810). Total phytosterol content was negatively correlated with total tocopherol content (r = −0.740, *p* < 0.01).

### 3.5. Cluster Analysis of Fruit Quality Traits in 30 C. retusus Germplasm Accessions

**Figure 2:**
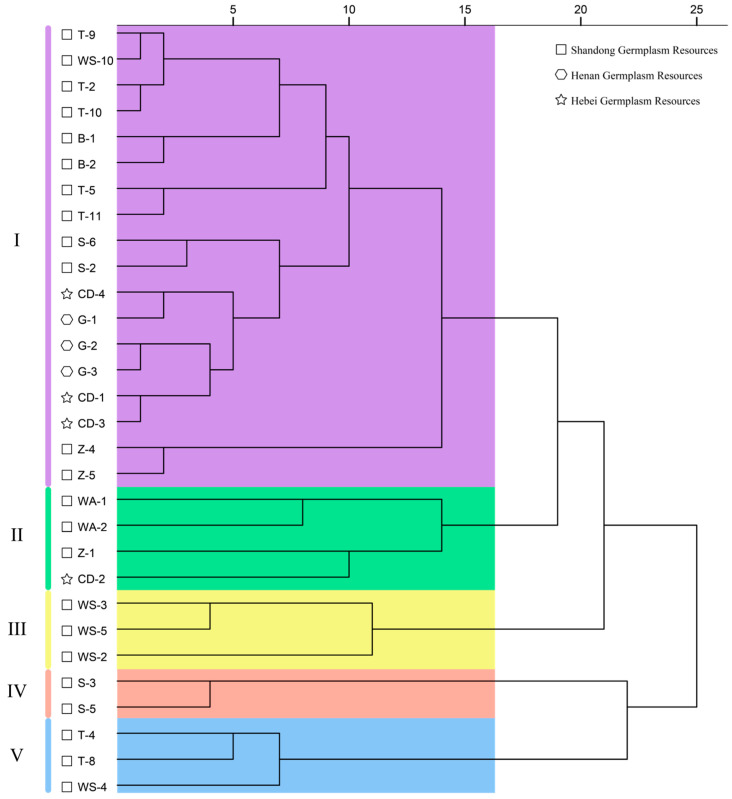
Hierarchical cluster analysis of fruit quality traits in different *C. retusus* germplasm accessions.

Cluster analysis of standardized data for fruit morphology, fatty acids, phytosterols, and tocopherols across 30 *C. retusus* accessions (Figure 2) revealed five distinct groups at a distance threshold of 16: Group I comprised eighteen accessions (T-9, WS-10, T-2, T-10, B-1, B-2, T-5, T-11, S-6, S-2, CD-4, G-1, G-2, G-3, CD-1, CD-3, Z-4, Z-5); Group II contained four (WA-1, WA-2, Z-1, CD-2); Group III consisted of three (WS-3, WS-5, WS-2); Group IV included two (S-3, S-5); and Group V encompassed three (T-4, T-8, WS-4). Analysis of provenance information indicated substantial variation, demonstrating that clustering did not strictly correspond to geographical origin.

### 3.6. Principal Component Analysis of Fruit Quality Traits in C. retusus Germplasm Accessions

Principal component analysis (PCA) was performed on the aforementioned traits across *C. retusus* germplasm accessions. Principal components with eigenvalues greater than 1 were extracted, as presented in Table 6. The results indicated that the first five principal components collectively accounted for 81.333% of the total variance, explaining the majority of the variation. Specifically, PC1 had an eigenvalue of 4.922, contributing 32.810% to the variance. This component was dominated by the traits fresh weight, FW, grain weight, GL, GW, kernel weight, and kernel rate, mainly reflecting information on various phenotypic traits of the fruit. PC2 (eigenvalue = 2.775, contribution rate = 18.502%) was primarily associated with SFA, MUFA, PUFA, and phytosterol content, mainly reflecting information on the lipids in *C. retusus* kernels. PC3 (eigenvalue = 2.050, contribution rate = 13.666%) was characterized mainly by FW, FSI, GL, kernel weight, and oil content, mainly expressing the plumpness of *C. retusus* fruits. PC4 (eigenvalue = 1.333, contribution rate = 8.884%) was dominated by the VD/GT ratio, mainly reflecting information on the yield of *C. retusus* fruits. PC5 (eigenvalue = 1.121, contribution rate = 7.471%) was primarily associated with SFA, MUFA, phytosterol, and total tocopherols, mainly reflecting information on secondary metabolites.

### 3.7. Comprehensive Assessment of Fruit Quality in 30 C. retusus Germplasm Accessions

Based on the eigenvalues and loading matrix of the five principal components, the principal component scores were calculated (Table 7). The comprehensive score (F) for each individual plant was then derived by combining these scores with the contribution rate weights of each principal component, using the following formula:

F = (0.32810F_1_ + 0.18502F_2_ + 0.13666F_3_ + 0.08884F_4_ + 0.07471F_5_)/0.81333 (1)

The F scores for the fruit quality traits of different germplasm accessions were calculated and ranked according to the results. The accessions WS-4, T-8, T-4, WS-33, and T-9, which ranked in the top five based on F scores, exhibited superior fruit quality traits and represent promising candidates for breeding with desirable fruit characteristics.

### 3.8. Analysis of Secondary Metabolite Composition in Kernels from 30 C. retusus Germplasm Accessions

**Figure 3:**
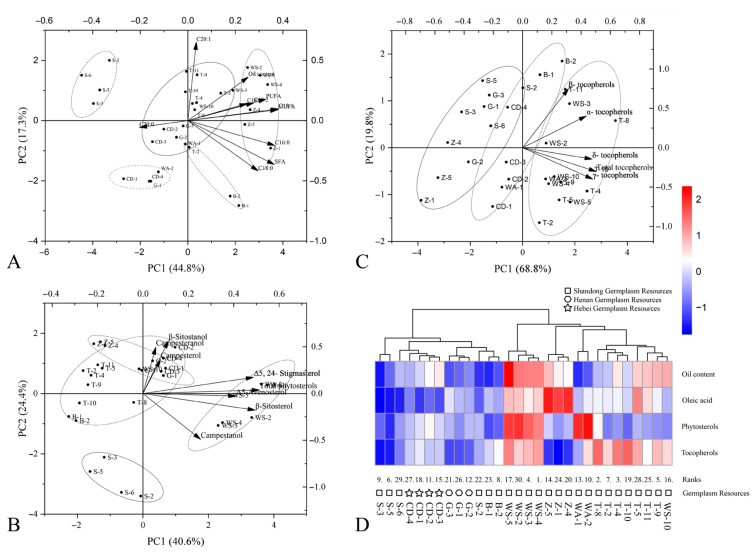
Principal component analysis and cluster analysis heatmap based on different kernel secondary metabolites. (**A**) Principal component analysis (PCA) based on fatty acid composition. (**B**) Principal component analysis (PCA) based on phytosterol composition. (**C**) Principal component analysis (PCA) based on tocopherol composition. (**D**) Cluster analysis heatmap of kernel secondary metabolites.

Analysis of numerous fruit morphological indicators was included in the aforementioned assessments to understand fruit quality characteristics across different germplasm accessions. Two-dimensional PCA biplots were constructed based on the composition and content data of fatty acids, phytosterols, and tocopherols in the kernels, as shown in Figure 3A–C, respectively. A clustered heatmap was generated using standardized data for oil content, oleic acid content, total sterols, and total tocopherols (Figure 3D).

For fatty acid composition and content, the first two PCs cumulatively explained 62.10% of the total variation. The 30 *C. retusus* accessions were divided into five clusters (Figure 3A). Cluster I was characterized by high oil content and high proportions of MUFAs and PUFAs, with prominent contributions from oleic acid (C18:1), linoleic acid (C18:2), and linolenic acid (C18:3). Cluster II was distinguished by high amounts of arachidic acid (C20:0) and eicosenoic acid (C20:1). The remaining clusters exhibited moderate levels across all parameters. Based on the analysis, Cluster I accessions (e.g., WS-2, WS-3, WS-5), combining high oil content with high linoleic and linolenic acid levels, represent ideal oil-type *C. retusus* germplasm resources.

For phytosterol composition and content, the first two PCs cumulatively explained 65.00% of the total variation. The germplasm accessions were classified into four clusters (Figure 3B). Cluster I featured high total phytosterols, β-sitosterol, campestanol, Δ5-avenasterol, and Δ5,24-stigmasterol. Clusters II and III were rich in campesterol, campesteranol, and β-sitostanol. Cluster IV showed no significant differences in sterol composition or content. PCA results indicated that accessions WA-1, WA-2, WS-2, WS-3, WS-4, and WS-5 possessed higher phytosterol contents, identifying them as candidate resources for functional oil development.

For tocopherol composition and content, the first two PCs cumulatively explained 88.60% of the total variation. The accessions were grouped into three clusters (Figure 3C). Cluster I exhibited significantly higher total tocopherols and α-, β-, γ-, and δ-tocopherol contents compared to other clusters. No significant differences were observed in the tocopherol compositions between Clusters II and III. Accessions T-4, T-5, T-8, T-10, WS-4, and WS-5, distinguished by their high tocopherol contents, are the preferred germplasm accessions for high-antioxidant-activity oil production.

The clustered heatmap of the four components (Figure 3D) revealed clustering patterns based on provenance, consistent with the PCA results (Figure 3A–C) but in contrast to the cluster analysis incorporating fruit phenotypic traits (Figure 2). Accessions of WS provenance (Qingzhou City, Shandong Province, China) generally exhibited superior performance across components, with significantly higher oil content and phytosterols; the accessions ranked 1st, 4th, 16th, 17th, and 30th in the PCA were all within this group. Accessions of Z provenance (Boshan District, Zibo City, Shandong Province, China) showed significantly higher oleic acid content. Accessions of WA provenance (Anqiu City, Shandong Province, China) demonstrated significantly higher phytosterol content. Accessions of T provenance (Mount Tai, Tai’an City, Shandong Province, China) exhibited relatively higher tocopherol content.

## 4. Discussion

### 4.1. Analysis of Fruit Quality in C. retusus Germplasm Accessions

*C. retusus* exhibits a broad geographical distribution [37] and demonstrates exceptional environmental adaptability [38], enabling its robust growth across diverse ecosystems. Exploiting the potential value of fringe tree oil may reduce reliance on long-distance transportation of edible oils.

In this study, the UFA content of *C. retusus* kernel oil was determined to be 88.51%. This is comparable to UFA levels in common woody oil crops such as olives (*Olea europaea*, 89.72%), tea oil (*Camellia oleifera*, 83.48%), and truncate-leaved maples (*Acer truncatum*, 91.71%) [6,46,47]. Oleic acid (C18:1) predominated among the UFAs, constituting 54.18% of the total fatty acids. As shown in Table 3, cultivars Z-1 (58.97%), Z-4 (59.43%), and Z-5 (59.67%) exhibited significantly higher oleic acid contents than other cultivars. Oleic acid reduces concentrations of total serum cholesterol and low-density lipoprotein cholesterol (LDL-C, “harmful” cholesterol) while maintaining high-density lipoprotein cholesterol (HDL-C, “beneficial” cholesterol) levels [13,14]. Linoleic acid (C18:2) was the second most abundant UFA, ranging from 15.33% to 30.35%. As a biosynthetic precursor to γ-linolenic acid and gondoic acid, particularly in the skin, linoleic acid is essential. Deficiency impairs skin barrier function, increasing transepidermal water loss [17]. Linoleic acid also reduces serum cholesterol and LDL-C concentrations [48,49]. Other detected fatty acids included α-linolenic acid (C18:3, 9.57%), arachidic acid (C20:0, 0.14%), and gondoic acid (C20:1). α-Linolenic acid serves as a precursor to docosahexaenoic acid (DHA), which is crucial for brain and neuronal development in infants and young children [17,18]. Collectively, these UFAs enhance immune function, mitigate pro-inflammatory factors, prevent infarction, and suppress allergic responses [17,18]. The ratio of SFA, MUFA, and PUFA in *C. retusus* kernel oil was approximately 6:55:33, resembling the profiles of rapeseed and olive oil. Thus, *C. retusus* kernel oil represents a promising edible vegetable oil source.

Phytosterols, triterpenoid metabolites structurally analogous to cholesterol, are widely distributed in plant roots, stems, leaves, and fruits but are primarily concentrated in vegetable oils. In oils, phytosterols exist predominantly (>50–97%) as free sterols, with β-sitosterol being the most abundant. In approximately 60–70% of vegetable oils, β-sitosterol comprises 50–80% of total phytosterols [46]. While some studies indicate dietary phytosterols lower LDL-C levels, others suggest they may inhibit the absorption and plasma concentrations of fat-soluble vitamins and antioxidants [50]. The U.S. Food and Drug Administration (FDA) has issued a qualified health claim linking plant sterol intake to reduced coronary heart disease risk, recommending a daily intake of ≥2 g [51]. The average phytosterol content in *C. retusus* oil was 279.58 mg/100 g. Seven phytosterols were identified, with β-sitosterol as the principal component. This phytosterol content significantly exceeded that of olive oil (*O. europaea*, 206.82 mg/100 g), shining-leaved yellowhorn oil *(Xanthoceras sorbifolium*, 185.3 mg/100 g), and peanut oil (*Arachis hypogaea*, 135 mg/100 g) [52,53,54], indicating its potential as a functional ingredient for phytosterol-enriched foods.

Tocopherols are viscous, pale yellow, oil-soluble compounds [55]. Among natural tocopherols, γ-tocopherol possesses the strongest antioxidant capacity [28]. Dietary vegetable oils are the primary source of tocopherols for humans, with the FDA recommending a daily intake of 30 mg for adults [27,56]. The γ-tocopherol content in *C. retusus* oil (520.97 µg/g) was significantly higher than in soybean oil (285 µg/g), olive oil (347.5 µg/g), and tea oil (280 µg/g) [34,57,58], suggesting superior inherent antioxidant activity and potentially enhanced oxidative stability. In their study on sunflower oil (*Helianthus annuus*), Abrante-Pascual et al. demonstrated that, under accelerated storage conditions (70 °C), sunflower oil supplemented with a 3% γ-tocopherol extract exhibited extended stability, reaching the peroxide value threshold (POV = 20 meq/kg) after 31 days. In contrast, the unsupplemented control reached this threshold within just 10 days [59]. Meanwhile, Zhang et al. demonstrated in their study on corn oil (*Zea mays*) that the experimental group supplemented with 200 mg/kg of a natural additive (phytosterol esters: tocopherols = 1:1) exhibited significantly superior antioxidant efficacy compared to BHA and BHT [60]. This suggests that *C. retusus* oil itself possesses strong inherent antioxidant properties, potentially allowing for a reduction in the amount of antioxidant additives required. Furthermore, its significantly higher γ-tocopherol content relative to other woody oil crops is notable, as this compound exerts anti-atherosclerotic and anti-aging effects by enhancing cellular antioxidant responses, regulating lipid metabolism, and reducing blood lipids [29,30,31]. This highlights the considerable potential of *C. retusus* oil for developing tocopherol-rich functional foods and antioxidant cosmetics.

### 4.2. Comprehensive Evaluation of Fruit Quality Traits in C. retusus Germplasm Accessions

Comprehensively analyzing *C. retusus* fruits alongside their oil content and secondary metabolites enables the preliminary screening of morphological indicators for evaluating kernel oil quality. Correlation analysis of 15 selected indicators (Figure 1) revealed a significant positive correlation between kernel dry weight and oil content (r = 0.760, *p* < 0.01), suggesting its potential as a key phenotypic marker for selecting high-oil-content accessions in *C. retusus* breeding. This finding contrasts with Liang et al. [6], who reported no correlation between oil content and any morphological traits in *Acer truncatum* kernels. This discrepancy may arise because *C. retusus* kernels (average dry weight 0.12 g) are smaller than those of *A. truncatum* (0.17 g), making kernel dry weight a critical limiting factor for oil accumulation in *C. retusus*. Cluster analysis of these 15 traits across the 30 accessions (Figure 2) showed no grouping based on provenance, highlighting complex variability in fruit quality traits. However, cluster analysis of the major secondary metabolites (Figure 3D) revealed distinct grouping by provenance, presenting an inconsistent pattern. Ji et al. [61] demonstrated that phenotypic traits are shaped by both genetic variation and environmental factors. This complex interplay contributes to greater phenotypic diversity. Consequently, breeding oil-type *C. retusus* cultivars could involve first selecting provenances with superior secondary metabolite profiles, followed by cultivating elite accessions from these provenances under optimal environmental conditions to develop varieties with desirable fruit traits like large kernel size and plumpness, ultimately enhancing oil yield.

Principal component analysis (PCA) reduces the dimensionality of fruit phenotypic and secondary metabolite traits, transforming them into fewer principal components to elucidate the relative importance of different indicators in comprehensive evaluation [62]. In this study, PCA and comprehensive evaluation based on 15 traits identified accessions WS-4, T-8, T-4, WS-3, and T-9 as having the top five F scores. Analysis of kernel secondary metabolites (Figure 3) revealed that WS-4 and WS-3 exhibited richness across multiple metabolite classes, while T-8, T-4, and T-9 had notably high total tocopherol content. Paradoxically, the elite WS provenance group (containing WS-2, WS-3, WS-4, WS-5, WS-10) displayed highly variable comprehensive evaluation ranks (30th, 4th, 1st, 17th, and 16th), encompassing both the best and worst performers, consistent with our earlier conclusions. This indicates that even accessions rich in secondary metabolites can exhibit significant yield variation due to suboptimal phenotypic morphology. To select *C. retusus* accessions with high overall evaluation scores and simultaneously high performance in oil content, phytosterols, and tocopherols, WS-4 and WS-3 were identified as promising candidates for oil-type germplasm development.

While this study provides foundational insights into the fatty acid composition and extraction potential of *C. retusus* kernel, two critical directions for future research are clearly indicated. Firstly, planned multi-year monitoring of kernel fatty acid profiles is essential to quantify and understand the inherent inter-annual variability driven by environmental factors, ensuring a robust characterization of this resource. Secondly, and crucially, comprehensive toxicological evaluation and assessment of consumer acceptability of the final extract are imperative prerequisites before any recommendation for human consumption can be made. Addressing both the consistency of the raw material and the safety of the end product will be the focus of our subsequent investigations, paving the way for responsible development and potential commercialization of *C. retusus* kernel-derived products.

## 5. Conclusions

The fruit oil of *Chionanthus retusus* (fringe tree) contains abundant components such as oleic acid, β-sitosterol, and γ-tocopherol, demonstrating potent antioxidant capacity. This suggests its potential to reduce reliance on synthetic antioxidant food additives. Furthermore, accessions WS-4 and WS-3 represent promising oil-bearing germplasm resources for *C. retusus*. These findings will facilitate the genetic improvement or selection of cultivars aimed at producing high-quality edible oil.

## Figures and Tables

**Table 1:** Geographic source of 30 *C. retusus* superior trees.

Superior Trees No.	Geographic Source	Location		Altitude (m)	Annual Precipitation (mm)	Annual Mean Temperature (°C)
		Latitude (°N)	Longitude (°E)			
T-2	Mount Tai, Tai’an City, Shandong Province	36°13′14″	117°7′34″	161	740	11.73
T-4	Mount Tai, Tai’an City, Shandong Province	36°13′14″	117°7′34″	161	740	11.73
T-5	Mount Tai, Tai’an City, Shandong Province	36°13′14″	117°7′34″	161	740	11.73
T-8	Mount Tai, Tai’an City, Shandong Province	36°13′14″	117°7′34″	161	740	11.73
T-9	Mount Tai, Tai’an City, Shandong Province	36°13′14″	117°7′34″	161	740	11.73
T-10	Mount Tai, Tai’an City, Shandong Province	36°13′14″	117°7′34″	161	740	11.73
T-11	Mount Tai, Tai’an City, Shandong Province	36°13′14″	117°7′34″	161	740	11.73
Z-1	Boshan District, Zibo City, Shandong Province	36°21′36″	117°59′16″	190	680	13.83
Z-4	Boshan District, Zibo City, Shandong Province	36°21′36″	117°59′16″	190	680	13.83
Z-5	Boshan District, Zibo City, Shandong Province	36°21′36″	117°59′16″	190	680	13.83
B-1	Boshan District, Zibo City, Shandong Province	36°30′58″	117°48′51″	190	680	13.83
B-2	Boshan District, Zibo City, Shandong Province	36°30′58″	117°48′51″	190	680	13.83
S-2	Yiyuan Country, Zibo City, Shandong Province	35°59′42″	118°12′59″	190	680	13.83
S-3	Yiyuan Country, Zibo City, Shandong Province	35°59′42″	118°12′59″	190	680	13.83
S-5	Yiyuan Country, Zibo City, Shandong Province	35°59′42″	118°12′59″	190	680	13.83
S-6	Yiyuan Country, Zibo City, Shandong Province	35°59′42″	118°12′59″	190	680	13.83
WS-2	Qingzhou City, Weifang City, Shandong Province	36°41′6″	118°18′7″	101	679	13.36
WS-3	Qingzhou City, Weifang City, Shandong Province	36°41′6″	118°18′7″	101	679	13.36
WS-4	Qingzhou City, Weifang City, Shandong Province	36°41′6″	118°18′7″	101	679	13.36
WS-5	Qingzhou City, Weifang City, Shandong Province	36°41′6″	118°18′7″	101	679	13.36
WA-1	Anqiu City, Weifang City, Shandong Province	36°12′2″	119°1′36″	101	679	13.36
WA-2	Anqiu City, Weifang City, Shandong Province	36°12′2″	119°1′36″	101	679	13.36
CD-1	Chengde City, Hebei Province	41°6′53″	117°48′18″	327	493	9.09
CD-2	Chengde City, Hebei Province	41°6′53″	117°48′18″	327	493	9.09
CD-3	Chengde City, Hebei Province	41°6′53″	117°48′18″	327	493	9.09
CD-4	Chengde City, Hebei Province	41°6′53″	117°48′18″	327	493	9.09
G-1	Tongbai City, Nanyang City, Henan Province	32°25′16″	113°17′25″	283	821	13.4
G-2	Tongbai City, Nanyang City, Henan Province	32°25′16″	113°17′25″	283	821	13.4
G-3	Tongbai City, Nanyang City, Henan Province	32°25′16″	113°17′25″	283	821	13.4

Note: Global geographic and climatic factors were obtained from WorldClim (http://www.worldclim.org (accessed on 23 September 2024)).

**Table 2:** Variation in morphological characteristics of 30 *Chionanthus retusus* accessions. Different superscript letters indicate significant differences among germplasms (*p* < 0.05). The same applies to subsequent tables.

Germplasm ID	Fruit Traits							Kernel Traits				
	Fresh Weight (Per 100 Grains)/g	Fruit Length/mm	Fruit Width/mm	Fruit Shape Index	Kernel Percentage/%	Kernel Weight (Per 100 Grains)/g	Shell Weight (Per 100 Grains)/g	Grain Weight (Per 100 Grains)/g	Grain Length/mm	Grain Transverse Diameter/mm	Shell Thickness/mm	Grain Length/Shell Thickness
T-2	79.36 ± 1.56 ^h^	12.26 ± 0.23 ^d^	10.38 ± 0.84 ^ab^	1.18 ^cd^	62.59 ^a^	14.99 ± 1.52 ^c^	8.96 ± 0.91 ^j^	23.95 ± 0.21 ^g^	10.56 ± 1.11 ^e^	7.14 ± 0.43 ^a^	0.38 ± 0.02 ^f^	30.03 ^a^
T-4	90.70 ± 3.81 ^e^	12.97 ± 1.12 ^cd^	9.42 ± 0.22 ^c^	1.39 ^b^	45.67 ^i^	14.74 ± 1.30 ^c^	17.53 ± 1.56 ^b^	32.27 ± 2,14 ^c^	11.64 ± 1.31 ^cd^	7.30 ± 0.55 ^a^	0.68 ± 0.09 ^bc^	16.56 ^jk^
T-5	47.42 ± 0.76 ^m^	10.90 ± 0.25 ^ef^	7.81 ± 0.05 ^e^	1.40 ^b^	54.07 ^d^	7.75 ± 0.54 ^j^	6.58 ± 0.71 ^l^	14.33 ± 1.11 ^k^	10.17 ± 1.04 ^e^	5.76 ± 0.72 ^bc^	0.39 ± 0.05 ^f^	24.47 ^cd^
T-8	121.30 ± 7.51 ^a^	15.37 ± 0.38 ^a^	11.38 ± 0.18 ^a^	1.35 ^b^	44.84 ^ij^	16.17 ± 1.83 ^ab^	19.90 ± 0.25 ^a^	36.07 ± 0.98 ^a^	13.57 ± 1.21 ^ab^	7.49 ± 0.67 ^a^	0.67 ± 0.10 ^c^	20.27 ^fg^
T-9	89.30 ± 2.79 ^e^	12.43 ± 0.56 ^d^	10.03 ± 0.47 ^b^	1.24 ^bc^	54 ^d^	14.22 ± 1.25 ^c^	12.11 ± 0.11 ^f^	26.33 ± 2.21 ^de^	10.46 ± 1.03 ^ef^	6.88 ± 0.52 ^ab^	0.55 ± 0.04 ^d^	20.53 ^fg^
T-10	69.33 ± 1.52 ^ij^	11.26 ± 0.43 ^e^	10.30 ± 0.93 ^ab^	1.09 ^e^	57.2 ^c^	11.52 ± 1.11 ^e^	8.62 ± 0.92 ^j^	20.15 ± 1.34 ^i^	9.60 ± 0.87 ^ef^	6.90 ± 0.29 ^ab^	0.43 ± 0.03 ^de^	25.09 ^c^
T-11	61.02 ± 0.34 ^k^	11.99 ± 0.25 ^de^	9.03 ± 0.71 ^c^	1.33 ^b^	49.53 ^g^	9.78 ± 0.99 ^fg^	9.97 ± 0.93 ^hi^	19.75 ± 1.23 ^i^	10.24 ± 1.98 ^e^	6.22 ± 0.54 ^ab^	0.51 ± 0.06 ^d^	21.14 ^f^
WS-2	49.98 ± 0.63 ^m^	9.53 ± 0.72 ^g^	7.81 ± 0.60 ^e^	1.23 ^c^	51.09 ^f^	8.16 ± 0.87 ^i^	4.93 ± 0.55 ^m^	15.97 ± 0.94 ^k^	7.51 ± 3.20 ^g^	5.12 ± 0.42 ^c^	0.45 ± 0.07 ^de^	19.68 ^fgh^
WS-3	73.85 ± 2.14 ^i^	11.83 ± 0.89 ^de^	9.70 ± 0.29 ^bc^	1.22 ^c^	58.4 ^b^	14.53 ± 1.32 ^c^	10.36 ± 1.31 ^h^	24.89 ± 2.12 ^f^	9.70 ± 1.21 ^ef^	7.15 ± 0.71 ^a^	0.43 ± 0.02 ^de^	25.58 ^bc^
WS-4	112.71 ± 3.58 ^b^	13.73 ± 0.98 ^c^	11.08 ± 1.01 ^a^	1.24 ^bc^	48.9 ^g^	17.08 ± 1.98 ^a^	17.84 ± 2.11 ^a^	34.92 ± 3.13 ^b^	11.65 ± 0.85 ^cd^	7.68 ± 0.66 ^a^	0.69 ± 0.08 ^bc^	18.35 ^i^
WS-5	60.92 ± 2.81 ^kl^	11.13 ± 1.05 ^e^	9.00 ± 1.20 ^cd^	1.24 ^bc^	57.09 ^c^	10.19 ± 1.05 ^f^	7.65 ± 0.45 ^k^	17.84 ± 1.34 ^j^	9.95 ± 0.48 ^ef^	6.31 ± 0.52 ^ab^	0.45 ± 0.02 ^de^	22.46 ^e^
WS-10	78.95 ± 2.14 ^h^	12.76 ± 0.89 ^d^	10.70 ± 0.29 ^ab^	1.19 ^c^	46.42 ^i^	11.23 ± 1.32 ^e^	8.06 ± 1.31 ^jk^	24.19 ± 2.12 ^gf^	9.32 ± 1.21 ^ef^	7.15 ± 0.71 ^a^	0.43 ± 0.02 ^de^	21.67 ^fc^
WA-1	72.75 ± 1.90 ^i^	15.36 ± 0.45 ^a^	9.23 ± 0.54 ^c^	1.67 ^a^	40.51 ^k^	10.12 ± 1.00 ^f^	14.85 ± 1.78 ^d^	24.97 ± 2.31 ^f^	14.32 ± 1.21 ^a^	6.39 ± 0.61 ^ab^	0.77 ± 0.13 ^b^	16.42 ^k^
WA-2	73.21 ± 1.05 ^i^	13.55 ± 0.21 ^c^	9.88 ± 1.21 ^bc^	1.38 ^b^	45.35 ^i^	11.09 ± 1.11 ^e^	13.36 ± 1.19 ^e^	24.45 ± 2.55 ^f^	12.09 ± 1.20 ^c^	7.40 ± 0.70 ^a^	0.60 ± 0.06 ^cd^	19.89 ^fg^
S-2	75.25 ± 2.06 ^i^	12.34 ± 0.59 ^d^	9.71 ± 0.76 ^bc^	1.27 ^bc^	45.93 ^i^	9.91 ± 0.78 ^fg^	13.65 ± 1.67 ^e^	23.56 ± 2.12 ^g^	10.34 ± 0.76 ^e^	6.49 ± 0.49 ^ab^	0.65 ± 0.04 ^c^	17.34 ^j^
S-3	100.76 ± 5.14 ^c^	11.68 ± 2.14 ^de^	11.19 ± 0.11 ^a^	1.04 ^e^	52.5 ^e^	14.68 ± 1.65 ^c^	17.28 ± 1.82 ^b^	31.96 ± 3.02 ^c^	9.42 ± 0.81 ^ef^	7.71 ± 0.95 ^a^	0.67 ± 0.07 ^bc^	17.21 ^j^
S-5	89.52 ± 3.84 ^e^	13.44 ± 0.78 ^c^	10.29 ± 1.00 ^ab^	1.31 ^b^	52.03 ^e^	16.67 ± 1.80 ^a^	15.07 ± 1.87 ^d^	31.74 ± 3.08 ^c^	11.74 ± 1.53 ^cd^	7.41 ± 0.83 ^a^	0.57 ± 0.02 ^cd^	21.08 ^f^
S-6	63.32 ± 1.81 ^k^	11.63 ± 0.37 ^de^	8.96 ± 0.51 ^cd^	1.30 ^b^	42.05 ^k^	9.53 ± 0.99 ^fg^	8.79 ± 0.88 ^j^	18.32 ± 1.51 ^j^	9.85 ± 0.49 ^ef^	6.12 ± 1.01 ^b^	0.51 ± 0.04 ^d^	20.44 ^fg^
B-1	49.24 ± 0.94 ^m^	11.62 ± 0.11 ^de^	8.74 ± 0.71 ^cd^	1.33 ^b^	47.58 ^h^	10.43 ± 1.21 ^ef^	11.49 ± 1.20 ^fg^	21.92 ± 1.93 ^h^	10.17 ± 1.10 ^e^	6.83 ± 1.14 ^ab^	0.42 ± 0.05 ^e^	24.82 ^cd^
B-2	70.55 ± 1.58 ^ij^	12.21 ± 0.17 ^d^	10.01 ± 1.11 ^b^	1.22 ^bc^	58.49 ^b^	15.95 ± 1.65 ^ab^	11.32 ± 1.10 ^fg^	27.27 ± 2.22 ^d^	9.88 ± 0.89 ^ef^	6.72 ± 0.72 ^ab^	0.42 ± 0.01 ^e^	26.79 ^b^
Z-1	58.14 ± 0.47 ^l^	14.13 ± 0.26 ^bc^	8.86 ± 0.39 ^cd^	1.60 ^a^	45.19 ^i^	8.95 ± 0.65 ^gh^	10.86 ± 1.03 ^gh^	19.81 ± 1.32 ^i^	12.67 ± 1.00 ^bc^	6.92 ± 0.53 ^ab^	0.45 ± 0.03 ^de^	26.12 ^b^
Z-4	83.89 ± 2.08 ^ef^	12.35 ± 0.62 ^d^	9.93 ± 0.64 ^bc^	1.24 ^bc^	39.9 l^k^	10.71 ± 1.09 ^ef^	16.13 ± 1.47 ^c^	26.84 ± 2.11 ^d^	10.85 ± 0.82 ^e^	6.95 ± 0.71 ^ab^	0.89 ± 0.09 ^a^	12.64 ^l^
Z-5	98.98 ± 0.56 ^cd^	12.48 ± 0.23 ^d^	11.60 ± 1.12 ^a^	1.07 ^e^	50.69 ^f^	12.53 ± 1.20 ^d^	12.19 ± 1.54 ^f^	24.72 ± 3.10 ^f^	9.72 ± 0.91 ^ef^	7.41 ± 0.45 ^a^	0.69 ± 0.08 ^bc^	17.65 ^ij^
CD-1	78.28 ± 0.56 ^h^	12.21 ± 0.23 ^d^	10.13 ± 1.41 ^ab^	1.21 ^bc^	56.15 ^c^	12.78 ± 1.24 ^d^	10.81 ± 1.52 ^gh^	22.76 ± 2.84 ^gh^	10.92 ± 0.83 ^e^	6.96 ± 0.65 ^ab^	0.49 ± 0.04 ^de^	22.29 ^e^
CD-2	72.55 ± 0.56 ^i^	13.76 ± 0.58 ^c^	9.53 ± 0.95 ^c^	1.44 ^b^	47.48 ^i^	11.47 ± 1.14 ^e^	12.64 ± 1.44 ^f^	24.16 ± 3.00 ^g^	13.27 ± 0.56 ^abc^	6.41 ± 0.42 ^ab^	0.43 ± 0.06 ^de^	30.86 ^a^
CD-3	80.79 ± 0.56 ^h^	11.42 ± 0.31 ^de^	11.05 ± 0.67 ^a^	1.03 ^ef^	55.61 ^d^	13.29 ± 1.07 ^d^	12.45 ± 1.23 ^f^	23.90 ± 3.34 ^g^	12.64 ± 0.72 ^bc^	6.08 ± 0.81 ^abc^	0.57 ± 0.08 ^cd^	22.18 ^e^
CD-4	66.41 ± 0.56 ^jk^	11.88 ± 0.37 ^de^	9,03 ± 0.95 ^cd^	1.32 ^b^	50.07 ^g^	10.33 ± 1.11 ^ef^	9.08 ± 1.31 ^j^	20.63 ± 3.61 ^i^	9.16 ± 0.80 ^ef^	6.21 ± 0.25 ^ab^	0.61 ± 0.05 ^bc^	15.02 ^k^
G-1	84.53 ± 0.56 ^ef^	12.62 ± 0.43 ^d^	10.87 ± 1.32 ^ab^	1.16 ^cd^	39.84 ^k^	10.64 ± 1.28 ^ef^	11.74 ± 1.44 ^fg^	26.71 ± 3.74 ^d^	10.48 ± 0.63 ^e^	5.96 ± 0.41 ^abc^	0.53 ± 0.07 ^d^	19.77 ^fgh^
G-2	86.24 ± 0.56 ^ef^	13.18 ± 0.33 ^cd^	11.71 ± 0.82 ^a^	1.13 ^cd^	53.11 ^d^	14.62 ± 1.34 ^c^	13.80 ± 1.56 ^e^	27.53 ± 2.10 ^d^	11.94 ± 0.79 ^cd^	6.31 ± 0.97 ^ab^	0.72 ± 0.03 ^b^	16.58 ^k^
G-3	81.44 ± 0.56 ^efg^	11.65 ± 0.27 ^de^	11.07 ± 0.88 ^a^	1.05 ^e^	52.22 ^e^	12.45 ± 1.20 ^d^	12.72 ± 1.54 ^f^	23.84 ± 3.11 ^g^	11.73 ± 0.47 ^cd^	5.81 ± 0.45 ^bc^	0.51 ± 0.08 ^de^	23.00 ^e^
Maximum	121.30	15.37	11.71	1.60	62.59	17.08	19.90	36.07	14.43	7.68	0.89	30.03
Minimum	47.42	9.53	7.81	1.03	39.84	7.75	4.93	14.33	7.51	5.12	0.38	12.64
Average	77.02 ± 17.69	12.46 ± 1.23	9.98 ± 1.03	1.26 ± 0.15	50.15 ± 5.83	12.22 ± 2.57	12.02 ± 3.47	24.33 ± 5.56	10.85 ± 1.47	6.71 ± 0.63	0.55 ± 0.13	21.20 ± 4.20
CV(%)	27.15	11.18	10.63	11.48	11.62	26.52	32.42	26.75	14.09	9.53	25.18	19.83

**Table 3:** Oil content and fatty acid composition of 30 *Chionanthus retusus* accessions.

Germplasm ID	Oil Content/%	Palmitic Acid (C16:0)	Stearic Acid (C18:0)	Oleic Acid (C18:1)	Linoleic Acid (C18:2)	Linolenic Acid (C18:3)	Arachidic Acid (C20:0)	Gondoic Acid (C20:1)	Saturated Fatty Acids (SFA)	Monounsaturated Fatty Acids (MUFA)	Polyunsaturated Fatty Acids (PUFA)
T-2	34.9 ± 0.65 ^ef^	1.28 ± 0.03 ^f^	1.16 ± 0.01 ^e^	54.63 ± 0.09 ^cd^	22.41 ± 0.02 ^gh^	9.17 ± 0.05 ^cde^	—	0.28 ± 0.04 ^i^	2.44 ± 0.07 ^cde^	54.91 ± 0.13 ^ef^	31.58 ± 0.07 ^hi^
T-4	38.2 ± 0.10 ^cd^	1.23 ± 0.12 ^f^	1.10 ± 0.01 ^e^	54.70 ± 0.17 ^cd^	22.44 ± 0.03 ^fg^	9.33 ± 0.09 ^cde^	—	0.35 ± 0.04 e^fg^	2.33 ± 0.31 ^de^	55.05 ± 0.21 ^de^	31.77 ± 0.12 ^hi^
T-5	39.2 ± 0.38 ^bcd^	1.25 ± 0.05 ^f^	1.34 ± 0.06 ^cd^	57.67 ± 0.25 ^b^	21.41 ± 0.03 ^gh^	11.47 ± 0.12 ^b^	—	0.38 ± 0.03 ^cd^	2.59 ± 0.14 ^cd^	55.05 ± 0.28 ^de^	32.88 ± 0.15 ^ghi^
T-8	34.5 ± 0.53 ^ef^	1.21 ± 0.12 ^f^	1.31 ± 0.09 ^cd^	54.73 ± 0.47 ^cd^	23.40 ± 0.05 ^efg^	8.60 ± 0.05 ^de^	—	0.38 ± 0.03 ^cd^	2.52 ± 0.24 ^cd^	55.11 ± 0.50 ^de^	32.00 ± 0.10 ^hi^
T-9	40.3 ± 0.87 ^bc^	1.22 ± 0.02 ^f^	0.98 ± 0.15 ^fg^	54.67 ± 0.45 ^cd^	24.42 ± 0.04 ^ef^	9.30 ± 0.17 ^cd^	—	0.38 ± 0.06 ^cd^	2.20 ± 0.21 ^def^	55.05 ± 0.51 ^de^	33.72 ± 0.21 ^fgh^
T-10	35.5 ± 0.60 ^ef^	1.21 ± 0.01 ^f^	1.04 ± 0.15 ^ef^	54.03 ± 0.08 ^cd^	23.48 ± 0.04 ^efg^	9.27 ± 0.16 ^cde^	—	0.38 ± 0.03 ^cd^	2.25 ± 0.19 ^def^	54.41 ± 0.11 ^efg^	32.75 ± 0.20 ^ghi^
T-11	39.6 ± 0.64 ^bcd^	1.06 ± 0.32 ^gh^	0.74 ± 0.08 ^gh^	55.77 ± 0.06 ^c^	23.40 ± 0.02 ^efg^	9.30 ± 0.55 ^cd^	—	0.35 ± 0.03 ^efg^	1.80 ± 0.42 ^g^	56.12 ± 0.09 ^de^	32.70 ± 0.52 ^ghi^
Z-1	37.8 ± 1.15 ^cd^	1.98 ± 0.06 ^ab^	1.52 ± 0.24 ^bc^	58.97 ± 0.28 ^ab^	25.47 ± 0.03 ^cde^	9.43 ± 0.11 ^cd^	—	0.32 ± 0.05 ^h^	3.50 ± 0.33 ^a^	59.29 ± 0.33 ^ab^	34.90 ± 0.14 ^fg^
Z-4	37.2 ± 0.96 ^cde^	1.28 ± 0.01 ^f^	1.53 ± 0.11 ^bc^	59.43 ± 0.04 ^ab^	26.48 ± 0.05 ^cd^	8.70 ± 0.06 ^de^	—	0.36 ± 0.09 ^e^	2.81 ± 0.16 ^bc^	59.79 ± 0.13 ^ab^	35.18 ± 0.11 ^f^
Z-5	40.0 ± 0.96 ^bc^	1.15 ± 0.01 ^fg^	1.64 ± 0.09 ^b^	59.67 ± 0.15 ^ab^	22.44 ± 0.04 ^fg^	9.40 ± 0.07 ^cd^	—	0.33 ± 0.03 ^gh^	2.79 ± 0.14 ^bc^	60.00 ± 0.18 ^a^	31.84 ± 0.11 ^de^
B-1	28.9 ± 0.58 ^h^	1.76 ± 0.01 ^cd^	1.88 ± 0.01 ^a^	53.60 ± 0.08 ^cd^	28.82 ± 0.04 ^b^	9.87 ± 0.35 ^cd^	—	0.27 ± 0.02 ^j^	3.64 ± 0.20 ^a^	53.87 ± 0.10 ^fgh^	38.69 ± 0.41 ^d^
B-2	30.5 ± 0.36 ^g^	1.69 ± 0.22 ^cde^	1.83 ± 0.05 ^ab^	53.10 ± 0.11 ^cd^	28.49 ± 0.06 ^b^	9.40 ± 0.06 ^cd^	—	0.28 ± 0.03 ^i^	3.52 ± 0.27 ^ab^	53.38 ± 0.14 ^fgh^	37.89 ± 0.12 ^de^
S-3	28.3 ± 0.35 ^h^	0.88 ± 0.04 ^h^	0.45 ± 0.01 ^hij^	49.53 ± 0.06 ^ih^	21.41 ± 0.03 ^gh^	8.27 ± 0.29 ^de^	0.13 ± 0.03 ^de^	0.34 ± 0.05 ^fgh^	1.33 ± 0.05 ^hi^	49.87 ± 0.11 ^jkl^	29.68 ± 0.32 ^ijk^
S-5	31.4 ± 0.95 ^g^	0.44 ± 0.08 ^i^	0.73 ± 0.08 ^gh^	50.17 ± 0.12 ^gi^	20.71 ± 0.03 ^ghi^	9.37 ± 1.15 ^cd^	0.13 ± 0.03 ^de^	0.35 ± 0.03 ^efg^	1.17 ± 0.16 ^ij^	50.52 ± 0.15 ^jkl^	30.08 ± 1.18 ^hij^
S-6	34.5 ± 1.60 ^ef^	0.41 ± 0.02 ^i^	0.72 ± 0.04 ^gh^	50.30 ± 0.06 ^gi^	20.39 ± 0.03 ^ghi^	5.00 ± 0.80 ^f^	0.13 ± 0.03 ^de^	0.40 ± 0.04 ^ab^	1.13 ± 0.06 ^ij^	50.70 ± 0.10 ^jkl^	25.39 ± 0.83 ^g^
S-2	29.2 ± 0.38 ^h^	0.42 ± 0.06 ^i^	0.58 ± 0.01 ^hi^	51.53 ± 0.37 ^fg^	24.41 ± 0.04 ^ef^	8.47 ± 0.81 ^de^	0.12 ± 0.03 ^ef^	0.39 ± 0.01 ^bc^	1.00 ± 0.07 ^jk^	51.92 ± 0.38 ^ij^	32.88 ± 0.85 ^ef^
WS-2	42.8 ± 1.01 ^b^	2.02 ± 0.31 ^ab^	0.80 ± 0.08 ^gh^	56.20 ± 0.04 ^bc^	29.38 ± 0.04 ^ab^	11.03 ± 1.01 ^b^	0.13 ± 0.03 ^de^	0.38 ± 0.04 ^cd^	2.82 ± 0.39 ^cd^	56.58 ± 0.08 ^ab^	40.41 ± 1.05 ^bc^
WS-3	42.4 ± 1.30 ^b^	1.63 ± 0.02 ^cde^	1.10 ± 0.09 ^e^	55.40 ± 0.08 ^bc^	25.34 ± 0.02 ^cde^	12.80 ± 0.51 ^a^	0.15 ± 0.03 ^c^	0.35 ± 0.03 ^efg^	2.73 ± 0.11 ^cde^	55.75 ± 0.11 ^bc^	38.14 ± 0.53 ^d^
WS-4	42.3 ± 0.49 ^b^	2.09 ± 0.02 ^ab^	1.09 ± 0.06 ^ef^	56.03 ± 0.06 ^bc^	30.34 ± 0.03 ^a^	12.40 ± 0.46 ^a^	0.13 ± 0.02 ^de^	0.37 ± 0.04 ^ef^	3.18 ± 0.08 ^bc^	56.40 ± 0.10 ^ab^	42.74 ± 0.49 ^a^
WS-5	47.5 ± 0.67 ^a^	1.80 ± 0.32 ^cd^	1.15 ± 0.11 ^e^	56.37 ± 0.12 ^bc^	30.35 ± 0.03 ^a^	11.43 ± 1.11 ^b^	0.13 ± 0.03 ^de^	0.36 ± 0.06 ^ef^	2.95 ± 0.43 ^bc^	55.73 ± 0.18 ^ab^	41.78 ± 1.14 ^ab^
WS-10	40.6 ± 0.62 ^bc^	1.06 ± 0.24 ^fg^	1.15 ± 0.07 ^e^	53.42 ± 0.10 ^cd^	18.36 ± 0.02 ^ijk^	13.06 ± 1.43 ^a^	0.13 ± 0.02 ^de^	0.36 ± 0.03 ^ef^	2.95 ± 0.49 ^bc^	55.73 ± 0.48 ^ab^	31.42 ± 127 ^bc^
WA-1	29.0 ± 0.45 ^h^	1.21 ± 0.02 ^f^	1.06 ± 0.01 ^ef^	53.53 ± 0.03 ^cd^	30.51 ± 0.05 ^a^	9.80 ± 0.10 ^d^	0.18 ± 0.04 ^b^	0.27 ± 0.04 ^j^	2.27 ± 0.03 ^def^	53.80 ± 0.07 ^fgh^	40.31 ± 0.15 ^bc^
WA-2	30.8 ± 1.00 ^g^	1.48 ± 0.03 ^e^	0.93 ± 0.09 ^fg^	54.63 ± 0.25 ^cd^	25.47 ± 0.04 ^cde^	5.03 ± 0.15 ^f^	0.21 ± 0.04 ^a^	0.26 ± 0.03 ^k^	2.41 ± 0.12 ^de^	54.89 ± 0.28 ^ef^	30.50 ± 0.19 ^hij^
CD-1	35.6 ± 0.73 ^ef^	1.27 ± 0.07 ^f^	1.04 ± 0.05 ^ef^	50.78 ± 0.57 ^gi^	15.33 ± 0.04 ^k^	9.05 ± 0.91 ^de^	—	0.25 ± 0.01 ^k^	2.24 ± 0.27 ^de^	51.03 ± 0.48 ^ijk^	24.38 ± 0.56 ^lm^
CD-2	37.2 ± 1.54 ^cd^	1.19 ± 0.05 ^f^	0.94 ± 0.07 ^fg^	51.77 ± 1.74 ^fg^	24.43 ± 0.02 ^ef^	10.08 ± 2.21 ^cd^	—	0.29 ± 0.01 ^i^	2.33 ± 0.94 ^de^	52.06 ± 0.56 ^ij^	34.51 ± 0.34 ^fg^
CD-3	38.2 ± 2.65 ^ef^	1.14 ± 0.07 ^f^	1.21 ± 0.09 ^de^	51.22 ± 0.25 ^fg^	19.37 ± 0.04 ^ijk^	10.24 ± 0.15 ^cd^	—	0.31 ± 0.03 ^k^	2.48 ± 0.52 ^de^	51.53 ± 0.28 ^ijk^	29.61 ± 0.19 ^ijk^
CD-4	34.2 ± 1.94 ^ef^	1.35 ± 0.03 ^f^	1.15 ± 0.10 ^e^	52.33 ± 0.73 ^fg^	15.34 ± 0.02 ^k^	9.67 ± 1.15 ^de^	—	0.27 ± 0.02 ^j^	2.79 ± 0.91 ^cde^	52.60 ± 0.28 ^hij^	25.01 ± 0.19 ^l^
G-1	31.3 ± 1.75 ^g^	1.31 ± 0.03 ^f^	1.24 ± 0.12 ^e^	53.54 ± 1.23 ^cd^	15.38 ± 0.06 ^k^	8.42 ± 1.48 ^de^	—	0.30 ± 0.03 ^i^	2.92 ± 0.63 ^bc^	53.84 ± 0.28 ^fgh^	23.80 ± 0.19 ^lm^
G-2	32.5 ± 1.83 ^g^	1.21 ± 0.07 ^f^	1.11 ± 0.11 ^ef^	53.21 ± 1.63 ^cd^	23.40 ± 0.04 ^efg^	9.82 ± 0.94 ^d^	—	0.32 ± 0.03 ^h^	2.67 ± 1.27 ^cd^	53.53 ± 0.28 ^fgh^	33.22 ± 0.19 ^fgh^
G-3	30.4 ± 1.41 ^g^	1.38 ± 0.03 ^f^	1.06 ± 0.05 ^ef^	54.38 ± 0.85 ^cd^	24.43 ± 0.05 ^ef^	10.06 ± 0.61 ^d^	—	0.33 ± 0.03 ^h^	2.26 ± 0.53 ^de^	54.71 ± 0.28 ^ef^	34.49 ± 0.19 ^fg^
Maximum	47.50	2.09	1.88	59.67	30.51	13.06	0.21	0.40	3.64	60.00	41.78
Minimum	28.30	0.41	0.45	49.53	15.33	5.00	—	0.26	1.00	49.87	23.80
Average	35.83 ± 4.87	1.29 ± 0.41	1.12 ± 0.33	54.18 ± 2.61	23.57 ± 4.19	9.57 ± 1.72	0.14 ± 0.03	0.33 ± 0.04	2.41 ± 0.64	54.51 ± 2.61	33.23 ± 4.83
CV/%	15.81	11.40	17.65	5.84	11.90	10.07	20.00	22.22	12.02	6.17	10.22

Note: Fatty acid composition is presented as relative percentage (%) of total fatty acids.

**Table 4:** Total phytosterol content and phytosterol composition of 30 *C. retusus* accessions.

Germplasm ID	Phytosterols mg/100 g							
	Campesterol	Campestanol	Campesteranol	β-Sitosterol	β-Sitostanol	Δ5-Avenasterol	Δ5,24-Stigmasterol	Total Sterols
T-2	11.41 ± 0.25 ^h^	15.25 ± 0.85 ^hi^	18.63 ± 0.31 ^ab^	166.19 ± 1.05 ^ef^	37.48 ± 0.94 ^de^	—	—	248.96 ± 0.93 ^de^
T-4	12.95 ± 0.57 ^fg^	12.09 ± 1.79 ^k^	16.74 ± 0.89 ^cd^	178.88 ± 1.98 ^de^	36.12 ± 0.74 ^e^	—	—	256.78 ± 0.56 ^cde^
T-5	11.49 ± 0.05 ^h^	12.76 ± 0.79 ^k^	19.50 ± 0.92 ^a^	187.63 ± 2.16 ^bcd^	35.18 ± 0.50 ^ef^	—	—	266.56 ± 0.78 ^cd^
T-8	14.13 ± 1.04 ^e^	28.10 ± 1.17 ^e^	13.44 ± 0.76 ^f^	195.13 ± 1.53 ^abc^	49.65 ± 0.64 ^a^	—	—	290.45 ± 1.93 ^c^
T-9	10.99 ± 0.05 ^hi^	17.53 ± 0.52 ^g^	15.92 ± 0.67 ^cd^	167.70 ± 2.91 ^def^	42.28 ± 0.20 ^b^	—	—	254.42 ± 1.25 ^cde^
T-10	11.98 ± 0.50 ^g^	23.57 ± 0.70 ^f^	14.96 ± 1.13 ^de^	155.93 ± 6.67 ^fg^	36.26 ± 0.16 ^e^	—	—	242.70 ± 1.06 ^de^
T-11	16.95 ± 0.13 ^d^	17.21 ± 0.28 ^g^	18.73 ± 0.58 ^ab^	174.53 ± 1.39 ^de^	32.57 ± 0.23 ^h^	—	—	259.99 ± 0.63 ^cde^
Z-1	22.03 ± 0.33 ^a^	17.58 ± 0.26 ^g^	19.33 ± 0.43 ^a^	163.85 ± 1.83 ^ef^	33.56 ± 0.89 ^g^	—	—	256.35 ± 0.31 ^cde^
Z-4	22.95 ± 0.81 ^a^	18.44 ± 0.98 ^g^	17.80 ± 0.47 ^bc^	170.28 ± 2.69 ^def^	38.60 ± 1.08 ^d^	—	—	268.07 ± 0.50 ^cd^
Z-5	21.93 ± 0.28 ^a^	16.88 ± 0.86 ^gh^	19.30 ± 0.26 ^a^	162.70 ± 2.00 ^ef^	33.88 ± 0.40 ^g^	—	—	274.69 ± 0.64 ^cd^
B-1	12.43 ± 0.15 ^g^	13.31 ± 0.80 ^k^	10.07 ± 0.41 ^h^	165.56 ± 2.76 ^ef^	33.74 ± 0.23 ^g^	—	—	235.11 ± 0.71 ^def^
B-2	12.18 ± 0.21 ^g^	14.21 ± 0.17 ^hij^	10.46 ± 0.55 ^gh^	172.60 ± 0.66 ^def^	31.66 ± 0.02 ^hi^	—	—	241.11 ± 0.88 ^de^
S-3	17.59 ± 0.30 ^c^	31.74 ± 1.56 ^cd^	13.94 ± 1.07 ^ef^	176.40 ± 0.46 ^de^	—	2.99 ± 0.44 ^de^	—	242.66 ± 1.62 ^de^
S-5	12.17 ± 0.14 ^g^	32.37 ± 0.39 ^c^	14.44 ± 0.28 ^de^	174.47 ± 3.32 ^de^	—	2.26 ± 0.46 ^de^	—	225.71 ± 1.21 ^ef^
S-6	11.83 ± 0.19 ^h^	32.84 ± 1.02 ^c^	11.75 ± 0.46 ^g^	198.70 ± 4.17 ^bc^	—	3.17 ± 0.09 ^de^	—	258.29 ± 0.84 ^cde^
S-2	14.43 ± 0.35 ^e^	36.60 ± 1.05 ^b^	10.95 ± 1.01 ^gh^	199.40 ± 1.91 ^bc^	—	5.97 ± 0.28 ^c^	—	267.35 ± 1.05 ^cd^
WS-2	11.90 ± 0.23 ^h^	36.28 ± 0.97 ^b^	15.75 ± 1.72 ^cd^	232.30 ± 4.69 ^a^	41.26 ± 0.65 ^bc^	7.92 ± 0.14 ^abc^	5.96 ± 0.46 ^c^	351.37 ± 0.54 ^a^
WS-3	11.95 ± 0.09 ^gh^	42.90 ± 5.30 ^a^	18.42 ± 1.08 ^ab^	224.67 ± 34.56 ^a^	32.59 ± 0.10 ^h^	2.43 ± 1.6 ^de^	4.46 ± 3.50 ^d^	337.42 ± 1.75 ^ab^
WS-4	12.43 ± 0.07 ^g^	31.89 ± 1.47 ^de^	15.46 ± 0.37 ^cde^	221.30 ± 2.61 ^a^	33.31 ± 0.29 ^g^	9.32 ± 0.37 ^a^	4.05 ± 0.58 ^e^	327.76 ± 1.83 ^ab^
WS-5	13.04 ± 0.18 ^f^	13.86 ± 1.20 ^jk^	15.24 ± 2.27 ^cde^	239.23 ± 3.43 ^a^	32.38 ± 0.11 ^h^	9.87 ± 0.31 ^a^	4.33 ± 0.32 ^d^	347.95 ± 1.43 ^a^
WS-10	19.13 ± 0.60 ^b^	14.82 ± 0.32 ^hij^	12.21 ± 1.17 ^fg^	154.78 ± 0.11 ^fg^	40.94 ± 2.11 ^bc^	9.13 ± 0.60 ^a^	4.82 ± 0.32 ^d^	252.21 ± 11.17 ^cde^
WA-1	20.39 ± 0.22 ^b^	28.19 ± 1.33 ^e^	15.75 ± 0.98 ^cd^	237.03 ± 2.25 ^a^	34.57 ± 0.10 ^f^	9.15 ± 0.21 ^a^	7.91 ± 0.64 ^ab^	352.99 ± 1.67 ^a^
WA-2	20.22 ± 0.58 ^b^	32.19 ± 1.13 ^c^	15.74 ± 0.17 ^cd^	240.67 ± 1.07 ^a^	39.52 ± 0.29 ^c^	3.75 ± 0.41 ^d^	8.50 ± 0.39 ^a^	360.59 ± 1.54 ^a^
CD-1	15.07 ± 0.32 ^de^	18.03 ± 0.24 ^g^	16.86 ± 1.06 ^cd^	183.58 ± 0.54 ^cde^	38.54 ± 1.90 ^d^	7.11 ± 0.45 ^b^	5.07 ± 0.32 ^cd^	278.03 ± 0.24 ^cd^
CD-2	20.65 ± 0.64 ^b^	19.01 ± 0.16 ^fg^	18.64 ± 9.05 ^ab^	197.52 ± 0.60 ^bc^	37.82 ± 4.05 ^de^	4.82 ± 0.32 ^cd^	4.65 ± 0.64 ^d^	291.01 ± 0.16 ^c^
CD-3	13.59 ± 0.70 ^f^	17.26 ± 0.30 ^g^	17.12 ± 6.41 ^bcd^	186.50 ± 0.33 ^cde^	38.47 ± 3.88 ^d^	6.93 ± 0.55 ^bc^	4.59 ± 0.70 ^d^	275.26 ± 0.30 ^cd^
CD-4	16.94 ± 1.54 ^cde^	16.77 ± 0.45 ^gh^	16.23 ± 5.55 ^cd^	181.64 ± 0.41 ^cde^	41.58 ± 3.27 ^bc^	6.48 ± 0.21 ^bc^	5.94 ± 1.54 ^c^	268.77 ± 0.45 ^cd^
G-1	14.60 ± 1.07 ^e^	15.84 ± 0.31 ^hi^	15.43 ± 4.84 ^cde^	190.32 ± 0.74 ^bcd^	38.19 ± 5.32 ^d^	8.43 ± 0.33 ^ab^	5.60 ± 1.07 ^c^	253.84 ± 0.31 ^cde^
G-2	16.22 ± 0.44 ^cde^	16.72 ± 0.57 ^gh^	18.67 ± 7.39 ^ab^	176.83 ± 0.68 ^de^	32.44 ± 4.44 ^h^	7.56 ± 0.52 ^b^	4.22 ± 0.44 ^d^	267.72 ± 0.57 ^cd^
G-3	15.34 ± 0.65 ^de^	18.61 ± 0.72 ^bcd^	16.89 ± 0.23 ^cd^	168.42 ± 2.73 ^def^	36.22 ± 2.04 ^e^	6.54 ± 0.15 ^bc^	3.82 ± 0.34 ^ef^	265.84 ± 9.32 ^cd^
Maximum	22.95	42.90	19.50	240.67	49.65	9.87	8.50	360.59
Minimum	10.99	12.09	10.07	154.78	—	—	—	225.71
Average	15.30 ± 3.59	22.10 ± 8.40	15.81 ± 2.63	188.16 ± 24.80	36.88 ± 4.04	6.32 ± 2.46	5.09 ± 1.49	279.58 ± 35.79
CV/%	27.22	22.55	18.77	14.92	12.54	14.45	16.19	15.68

**Table 5:** Total tocopherol content and tocopherol composition of 30 *C. retusus* accessions.

Germplasm ID	Tocopherols µg/g				
	α-Tocopherol	β-Tocopherol	γ-Tocopherol	δ-Tocopherol	Total Tocopherols
T-2	32.82 ± 0.45 ^def^	5.75 ± 0.11 ^efg^	555.60 ± 10.45 ^bcd^	11.65 ± 0.63 ^bcd^	605.82 ± 2.37 ^bcd^
T-4	37.53 ± 0.22 c	7.33 ± 0.32 ^d^	586.55 ± 14.76 ^a^	14.12 ± 0.87 ^a^	645.53 ± 5.84 ^a^
T-5	33.86 ± 0.83	6.85 ± 0.17 ^de^	572.71 ± 4.56 ^ab^	11.94 ± 0.42 ^bc^	625.36 ± 4.28 ^abc^
T-8	44.21 ± 1.56 ^a^	9.16 ± 0.23 ^ab^	583.85 ± 8.39 ^a^	14.88 ± 0.61 ^a^	652.10 ± 8.56 ^a^
T-9	36.38 ± 1.05 ^cde^	7.00 ± 0.61 ^de^	560.84 ± 5.28 ^abc^	12.24 ± 0.70 ^ab^	616.46 ± 6.10 ^abc^
T-10	40.19 ± 0.74 ^b^	7.83 ± 0.30 ^cd^	593.94 ± 13.92 ^a^	12.26 ± 0.61 ^ab^	654.22 ± 1.94 ^a^
T-11	39.64 ± 0.92 ^bc^	9.11 ± 0.45 ^ab^	524.26 ± 15.21 ^cd^	12.61 ± 0.94 ^ab^	585.62 ± 5.45 ^bcd^
Z-1	19.13 ± 0.60 ^h^	4.82 ± 0.32 ^fg^	452.21 ± 11.17 ^fg^	4.78 ± 0.11 ^i^	480.94 ± 2.11 ^efg^
Z-4	21.20 ± 0.67 ^h^	6.93 ± 0.55 ^de^	465.27 ± 6.82 ^efg^	4.96 ± 0.20 ^i^	498.36 ± 4.06 ^def^
Z-5	15.12 ± 0.59 ^i^	6.48 ± 0.21 ^a^	472.14 ± 9.76 ^efg^	5.25 ± 0.16 ^i^	501.99 ± 3.17 ^def^
B-1	44.15 ± 0.62 ^a^	8.43 ± 0.33 ^bc^	508.31 ± 7.24 ^cde^	8.03 ± 0.44 ^g^	568.92 ± 4.85 ^cde^
B-2	42.64 ± 0.71 ^ab^	9.56 ± 0.52 ^a^	523.63 ± 8.18 ^cd^	10.02 ± 0.53 ^def^	585.85 ± 1.81 ^bcd^
S-3	32.37 ± 0.61 ^ef^	6.54 ± 0.15 ^def^	439.49 ± 11.34 ^fgh^	6.54 ± 0.47 ^h^	484.94 ± 1.79 ^efg^
S-5	34.83 ± 0.38 ^de^	7.56 ± 0.41 ^cd^	438.73 ± 6.23 ^fgh^	8.51 ± 0.58 ^g^	489.63 ± 5.72 ^efg^
S-6	30.13 ± 0.45 ^g^	7.37 ± 0.50 ^cd^	484.36 ± 8.42 ^def^	7.67 ± 0.36 ^gh^	529.53 ± 6.44 ^def^
S-2	33.58 ± 0.79 ^de^	8.84 ± 0.39 ^bc^	498.91 ± 6.71 ^def^	9.25 ± 0.30 ^efg^	550.58 ± 5.13 ^cde^
WS-2	35.07 ± 0.32 ^de^	8.03 ± 0.24 ^bcd^	556.86 ± 7.06 ^abc^	8.58 ± 0.54 ^g^	608.54 ± 1.90 ^bcd^
WS-3	40.65 ± 0.64 ^b^	9.01 ± 0.16 ^ab^	558.64 ± 9.05 ^abc^	9.52 ± 0.60 ^efg^	617.82 ± 4.05 ^abc^
WS-4	33.59 ± 0.70 ^de^	7.26 ± 0.30 ^cde^	579.12 ± 6.41 ^a^	8.50 ± 0.33 ^g^	628.47 ± 3.88 ^abc^
WS-5	36.94 ± 1.54 ^cde^	6.77 ± 0.45 ^de^	586.23 ± 5.55 ^a^	11.64 ± 0.41 ^bcd^	641.58 ± 3.27 ^a^
WS-10	32.03 ± 0.33 ^de^	7.58 ± 0.26 ^cd^	569.33 ± 0.43 ^a^	11.85 ± 1.83 ^bcd^	603.56 ± 0.89 ^bc^
WA-1	30.60 ± 1.07 ^g^	5.84 ± 0.31 ^efg^	501.43 ± 4.84 ^cd^	10.32 ± 0.74 ^de^	548.19 ± 5.32 ^cde^
WA-2	36.22 ± 0.44 ^cde^	6.72 ± 0.57 ^de^	548.67 ± 7.39 ^bcd^	10.83 ± 0.68 ^cde^	602.44 ± 4.44 ^bcd^
CD-1	31.41 ± 0.25 ^g^	5.25 ± 0.85 ^efg^	518.63 ± 0.31 ^cde^	6.19 ± 1.05 ^h^	577.48 ± 0.94 ^cd^
CD-2	32.95 ± 0.57 ^de^	6.09 ± 1.79 ^efg^	516.74 ± 0.89 ^cde^	8.88 ± 1.98 ^g^	562.12 ± 0.74 ^cde^
CD-3	31.49 ± 0.05 ^g^	6.76 ± 0.79 ^ef^	519.50 ± 0.92 ^cde^	7.63 ± 2.16 ^gh^	563.18 ± 0.50 ^cde^
CD-4	34.13 ± 1.04 ^de^	8.10 ± 1.17 ^bcd^	513.44 ± 0.76 ^cde^	5.13 ± 1.53 ^i^	564.65 ± 0.64 ^cde^
G-1	30.99 ± 0.05 ^g^	7.53 ± 0.52 ^cd^	465.92 ± 0.67 ^efg^	7.70 ± 2.91 ^gh^	510.28 ± 0.20 ^def^
G-2	31.98 ± 0.50 ^g^	5.57 ± 0.70 ^efg^	474.96 ± 1.13 ^efg^	5.93 ± 6.67 ^h^	516.26 ± 0.16 ^de^
G-3	36.95 ± 0.13 ^cde^	7.21 ± 0.28 ^cd^	458.73 ± 0.58 ^fg^	7.53 ± 1.39 ^gh^	508.57 ± 0.23 ^ef^
Maximum	44.21	9.11	593.94	14.88	654.22
Minimum	15.12	4.82	438.73	4.78	480.94
Average	33.76 ± 6.43	7.24 ± 1.18	520.97 ± 47.47	9.16 ± 2.75	571.13 ± 53.70
CV/%	22,17	17.07	9.47	31,77	10.14

**Table 6:** Principal component analysis (PCA) of fruit traits in 30 *C. retusus* germplasm resources.

Traits	Principal Components (PC)				
	1	2	3	4	5
Fresh weight	0.178	0.016	0.170	−0.109	0.059
FW	0.150	0.125	−0.234	0.060	−0.070
FSI	−0.001	0.157	−0.382	0.071	0.039
Grain weight	0.192	0.030	0.103	−0.005	0.005
GL	0.120	0.103	−0.286	0.139	0.028
GW	0.145	0.064	0.136	0.105	−0.117
VD	−0.060	0.036	0.004	0.660	−0.145
Kernel weight	0.155	0.014	0.231	0.228	0.040
Kernel rate	0.190	0.033	−0.039	−0.123	0.022
Oil content	−0.080	0.199	0.218	−0.066	0.133
SFA	−0.044	0.221	0.072	0.059	−0.486
MUFA	−0.036	0.244	0.082	−0.277	−0.412
PUFA	−0.027	0.267	0.031	0.010	0.023
Phytosterol mg	−0.030	0.229	−0.064	−0.193	0.476
Total tocopherols	−0.038	0.174	0.168	0.275	0.439
Eigenvalue	4.922	2.775	2.050	1.333	1.121
Contribution rate (%)	32.810	18.502	13.666	8.884	7.471
Cumulative contribution rate (%)	32.810	51.312	64.978	73.862	81.333

**Table 7:** Comprehensive scores (F) of fruit quality traits in 30 *C. retusus* germplasm resources.

NO.	Principal Component Score					F Scores	Ranks
	F1	F2	F3	F4	F5		
WS-4	1.51596	2.09272	1.36305	−0.34555	0.57238	1.33147	1
T-8	2.24169	1.14722	−0.01586	0.49376	0.69923	1.280788	2
T-4	1.12543	0.531	0.358	−0.15575	0.59919	0.672984	3
WS-3	−0.3522	1.14704	1.176	0.6128	0.71118	0.448708	4
T-9	0.24627	0.18168	0.87142	0.16868	0.35447	0.338082	5
S-5	1.49493	−1.33189	−0.3518	0.65656	0.12274	0.323965	6
T-2	−0.05712	−0.16646	0.80731	1.97169	−0.52507	0.241865	7
B-2	0.05508	0.05648	0.65429	1.67989	−1.22328	0.216122	8
S-3	1.38769	−2.00214	0.90201	−0.62671	0.2812	0.213296	9
WA-2	0.37248	0.65631	−1.02351	−0.23319	1.00006	0.19398	10
CD-2	0.08004	0.44214	−1.41426	1.87668	0.34656	0.132052	11
G-2	0.68922	−0.39802	−0.06349	−0.68584	−0.39746	0.065407	12
WA-1	0.66734	1.26569	−3.06885	−0.7231	1.05342	0.059275	13
Z-5	0.27237	0.28316	1.27042	−1.70987	−1.61776	0.052386	14
CD-3	−0.00897	−0.67567	0.39362	0.35875	0.56125	−0.00044	15
WS-10	−0.37951	0.19109	0.91748	0.1539	−0.68416	−0.00151	16
WS-5	−1.50018	1.51688	0.73461	−0.1742	1.20883	−0.04468	17
CD-1	0.04012	−0.91603	0.07991	0.62953	0.64957	−0.05034	18
T-10	−0.73141	−0.45018	1.03498	1.07417	0.16342	−0.09123	19
Z-4	0.37528	0.46615	0.12362	−2.49828	−1.4951	−0.13201	20
G-3	−0.04337	−0.76749	−0.04268	−0.18696	−0.5495	−0.27015	21
S-2	0.10542	−1.22841	−0.78898	−0.74678	1.14105	−0.34623	22
B-1	−0.65094	0.02443	−0.43194	1.02443	−1.68655	−0.37265	23
Z-1	−0.40033	1.20091	−1.91016	0.28417	−2.76518	−0.43223	24
T-11	−0.84981	−0.13058	−0.2986	−0.2989	0.23186	−0.43405	25
G-1	0.05024	−1.12709	−0.25769	−0.63757	−1.0383	−0.44444	26
CD-4	−0.67125	−0.93193	−0.29619	−0.93952	0.05132	−0.63046	27
T-5	−1.84504	0.04488	−0.58909	0.50691	0.16995	−0.7621	28
S-6	−0.76152	−1.78822	−0.80252	−0.18202	0.98794	−0.77797	29
WS-2	−2.4679	0.66633	0.66887	−1.34767	1.07676	−0.7799	30

## Data Availability

The datasets generated during and/or analyzed during the current study are available from the corresponding author on reasonable request.

## Abbreviations

The following abbreviations are used in this manuscript:

BHA	Butylated hydroxyanisole
BHT	Butylated hydroxytoluene
EFAs	Essential fatty acids
LDL	Low-density lipoprotein
HDL	High-density lipoprotein
EPA	Eicosapentaenoic acid
DHA	Docosahexaenoic acid
UV	Ultraviolet
FL	Fruit length
FW	Fruit width
FSI	Fruit shape index
GL	Grain length
GW	Grain width
ST	Shell thickness
Kp	Kernel percentage
GC	Gas chromatograph
CV	Coefficient of variation
UFAs	Unsaturated fatty acids
SFA	Saturated fatty acids
MUFA	Monounsaturated fatty acids
PUFA	Polyunsaturated fatty acids
PCA	Principal component analysis
PC	Principal components
